# Socioeconomic variation in adherence to follow-up after an abnormal screening mammogram in the Danish breast cancer screening program

**DOI:** 10.1007/s10549-026-08014-3

**Published:** 2026-07-09

**Authors:** C. M. B. Lundorff, S. H. Njor, S. L. Madsen, S. F. Jørgensen

**Affiliations:** 1 Research Unit for Screening and Epidemiology, Lillebaelt Hospital, Beriderbakken 4, Vejle, 7100 Denmark; 2 Department of Regional Health Research, Southern Denmark University, Vejle, Denmark; 3 Department of Radiology, University Hospital of Southern Denmark, Odense, Denmark

**Keywords:** Socioeconomic position, Follow-up adherence, Breast cancer screening, Health inequality, Denmark

## Abstract

**Objectives:**

Timely and guideline-consistent follow-up after abnormal mammography findings is essential for early diagnosis and improved breast cancer outcomes. While socioeconomic position (SEP) is known to affect participation in screening, less is known about whether SEP also shapes women’s adherence to follow-up once an abnormal result is identified. We aimed to investigate socioeconomic and regional differences in the timeliness and adequacy of follow-up after abnormal screening mammograms in Denmark.

**Methods:**

We included women aged 50–69 years with an abnormal screening result in Denmark, 2016–2021. Follow-up was classified as timely if initiated within 30 days and as adequate if aligned with national guidelines. SEP indicators were income, wealth, education, employment, cohabitation, country of origin, and comorbidity. Risk ratios (RR) were estimated using binomial generalized linear models.

**Results:**

Among 35,087 women, 99.3% received follow-up within six months, and 96.2% had guideline-consistent follow-up. Delays varied regionally, with higher risks in the Capital, Northern, and Zealand Regions. Delayed follow-up was more common among non-Western immigrants (RR = 1.445, 95% CI: 1.298–1.609), unemployed women (RR = 1.084, 95% CI: 1.009–1.165), and those living alone (RR = 1.073, 95% CI: 1.014–1.135). Low SEP increased the risk of no follow-up within six months, particularly among women with low wealth, low education, or immigrant background.

**Conclusions:**

Although adherence was high, regional and socioeconomic disparities persist. Women with low SEP, immigrants, unemployed, and those living alone face higher risks of delayed or inadequate follow-up, highlighting a need to reduce inequalities in the diagnostic pathway.

**Supplementary Information:**

The online version contains supplementary material available at 10.1007/s10549-026-08014-3.

## Introduction

Breast cancer is the most common cancer among women worldwide [1]. It accounts for approximately 23% of all female cancer cases in Denmark [2]. While participation in mammography screening can reduce breast cancer mortality [3, 4], adherence to recommended follow-up of screen-detected abnormalities is critical for ensuring optimal outcomes and prognosis [5, 6].

Socioeconomic position (SEP) is known to influence participation in mammography screening [7–9]. Specifically, women with lower SEP, are less likely to participate in screening, and have higher mortality [10]. This may be due to several factors, including less frequent use of screening services, delays in diagnosis, and more advanced disease stages at diagnosis [11–13], as well as a higher burden of comorbid conditions among women with lower SEP [14–16].

It remains uncertain whether women with low SEP are also more likely to experience suboptimal diagnostic follow-up, including non-timely follow-up and non-adherence to recommended diagnostic procedures. In Denmark, individuals enter a structured cancer pathway following an abnormal screening result. This pathway is designed to ensure that women receive standardized follow-up care until a definitive diagnosis is made, whether it involves a false-positive finding or a screen-detected cancer (SDC) [17]. Thus, the decision of the appropriate diagnostic follow-up should not be subject to individual choice. However, despite this standardized approach, women with lower SEP consistently exhibit poorer prognoses following a cancer diagnosis [10, 13]. This raises the question of whether potential differences in pre-diagnostic processes, including screening follow-up, may contribute to these disparities.

To our knowledge, no studies have specifically evaluated whether SEP is associated with adherence to follow-up recommendations, including both the timing and proper completion of follow-up after abnormal mammography findings. Given the role of timely follow-up in achieving early diagnosis and favourable outcomes, this study seeks to fill an important gap, which is essential for identifying disparities in cancer care and developing interventions aimed at improving health equity. The aim of this study is to investigate whether SEP is associated with the non-adherence to follow-up after breast cancer screening and to identify which SEP variables are are most strongly associated with non-adherence.

## Methods

### Setting

In Denmark, a nationwide biennial mammography screening program for women aged 50–69 was implemented in 2007. The program became fully established nationwide in 2010, although some regions of Denmark had offered screening since the 1990s [18].The program is organized and administered by the five Danish regions, following national guidelines for breast cancer screening [19].

The Danish Breast Cancer Cooperative Group (DBCG) and the Steering Committee of the Danish Mammography Screening Database have published national clinical guidelines for the follow-up procedure, for woman with an abnormal screening mammography [19, 20].

According to these guidelines, all women with screen-detected abnormalities are recommended to undergo supplemental imaging, including a mammogram or tomosynthesis with additional projections. In addition, an ultrasound and a clinical examination should be performed—preferably during the same appointment. If relevant a biopsy should be taken from any suspicious areas. These three-four components are collectively referred to as a “diagnostic mammography”. In Denmark, all screening and follow-up procedures are provided free of charge for participating women.

### Study design and population

We included women aged 50–69 years who were invited for mammography screening between January 1, 2016, and December 31, 2021, and had an abnormal screening result. Women with more than one abnormal mammogram during the study period were included only once, using the first abnormal result. Women with a prior diagnosis of breast cancer were excluded. Additionally, women who died or emigrated within 30 days of screening were excluded.

### Definitions

Invasive breast cancers (DC50*) and carcinomas in situ (DD05*) detected within six months of the initial screening, were considered SDCs. In Denmark it is legally required that women must be offered an initial diagnostic procedure within 14 days after two radiologists reached consensus on a suspicious screening result (i.e. an abnormal screening). Furthermore, it is recommended that results are received within 10 working days, a target that is monitored [19, 20]. Considering these two recommendations, we defined the first follow-up test to be timely if performed within 30 days of screening. Diagnostic resolution was defined as a diagnosis of a SDC, or no cancer diagnosis within six months, i.e. false-positive screening.

We included all follow-up mammograms, breast tomosynthesis, ultrasounds, breast MRIs, and other imaging procedures. Additionally, we included biopsies (stereotactic or ultrasound-guided), surgeries, and other excisional procedures where the pathology topography was breast tissue.

Women were categorized according to follow-up tests received before resolution: (1) no follow-up within 6 months (2), mammography/tomosynthesis/MRI, ultrasound and biopsy (fine needle or core) (3), mammography/tomosynthesis/MRI and ultrasound only, and (4) less than expected, comprising individualized strategies not aligned with the recommended triple test (e.g. ultrasound or mammography alone). Groups 2 and 3 followed national guidelines [19, 20]. Each woman was assigned to one category based on the hierarchy of procedures recorded in the registry.

In addition, women were classified by timeliness of the first follow-up according to Danish Cancer Care Pathway regulations [17]. Non-timely follow-up was defined as occurring more than 30 days after screening mammography. A subgroup of women with false-positive screening results and biopsy was further analyzed for socio-economic differences compared to true positives.

Information on screening invitations was obtained from the Danish Quality Database for Mammography Screening (DKMS) [18]. Data on screening outcomes and diagnostic procedures were retrieved from the Danish National Patient Register [21]. Biopsy and surgery specimens, including cytological and histological diagnoses, were obtained from the National Pathology Register [22]. Cancer diagnoses were sourced from the Danish Cancer Register [23] and the Pathology Register. Age was retrieved as birthdates from the Civil Registration System [24].

Information on SEP was collected from the Income Register, Population Register, Education Register, and Work Classification Register at Statistics Denmark [25]. These registers provided data on income, assets, country of origin, cohabitation status, education level, employment status and severity of comorbidity.

All data were linked using the unique personal identification numbers mandatory for all Danish residents.

Income was calculated as the average equalized disposable income over the three years preceding the abnormal screening. Assets were determined by subtracting total debt from total assets at the beginning of the screening year and then averaged over the three prior years—except for 2016, where a two-year average was used due to data limitations. Income and assets were divided into quartiles and combined into a single variable, ‘wealth,’ categorized into levels.

Country of origin was categorized as Native, Western, or Non-Western. Cohabitation status was classified as either cohabiting or living alone at the start of the year prior to the abnormal mammogram. The highest level of education was classified based on the Danish ISCED-15 and combined based on the number of years of schooling: less than 10 years (ISCED 1–2), short education (11–14 years) (ISCED 3–4), bachelor’s degree (15–17 years) (ISCED 5), or master’s degree (more than 17 years) (ISCED 6–8) [26]. Employment status was retrieved for the year preceding the abnormal screening result and classified into 3 groups: employed, unemployed or retired. Employment was defined as working ≥ 6 months that year.

The women’s comorbidities were categorized as none, mild and moderate to severe based on Charlsons comorbidity Index score [27].

### Statistical analysis

Variation in timely follow-up across socioeconomic groups was presented as numbers and percentages and assessed using the Chi-squared test. Associations between SEP and adherence to follow-up were analyzed using log-binomial regression to estimate relative risks (RRs). Separate models were fitted for each SEP indicator (education, wealth, employment), with adjustment sets defined a priori using directed acyclic graphs (DAGs) [28].

SEP variables were analyzed separately and not mutually adjusted, as they represent distinct but interrelated dimensions; simultaneous adjustment was avoided to reduce overadjustment and control for mediating pathways [29, 30].

DAGs included SEP variables, age, region, cohabitation status, country of origin, and comorbidity, and guided covariate selection: region (wealth, comorbidity, age), education (age, country of origin, region), wealth (employment, age, country of origin, region), employment (age, education, comorbidity), cohabitation (age), country of origin (education, age), and comorbidity (age).

Interactions were assessed using multiplicative terms between correlated variables (e.g., education, employment, income, comorbidity, age).

Reference categories for SEP variables and region were chosen a-priori based on representativeness and statistical stability. Specifically, the largest or most representative groups were used as reference categories to ensure robust and meaningful comparisons. Therefore, comparisons should be interpreted relative to each specific reference group rather than as a uniform socioeconomic gradient across all variables.

Cumulative incidence curves illustrated time to first follow-up by region and screening outcome. Women were followed from abnormal screening to first follow-up within six months. Median time to follow-up by pathways and SEP is reported in Supplementary Material. Different sensitivity analyses were performed: (1) adjusting for prior abnormal findings (2), stratified by period (2016–2019 vs. 2020–2021) to assess COVID-19 impact, and (3) testing of an alternative pathway definition including ultrasound and biopsy as guideline-adherent follow-up.

Women with missing information on one or more socioeconomic variables were excluded using a complete-case approach, as missing SEP variables not only affects the factor of interest but also potential adjustments. As analyses were exploratory with multiple comparisons, p-values should be interpreted cautiously due to the potential risk of type 1 error inflation.

Statistical significance was set at a two-sided 5% level. Analyses were conducted in Stata v19.5 MP (StataCorp, College Station, TX, USA).

**Fig. 1:**
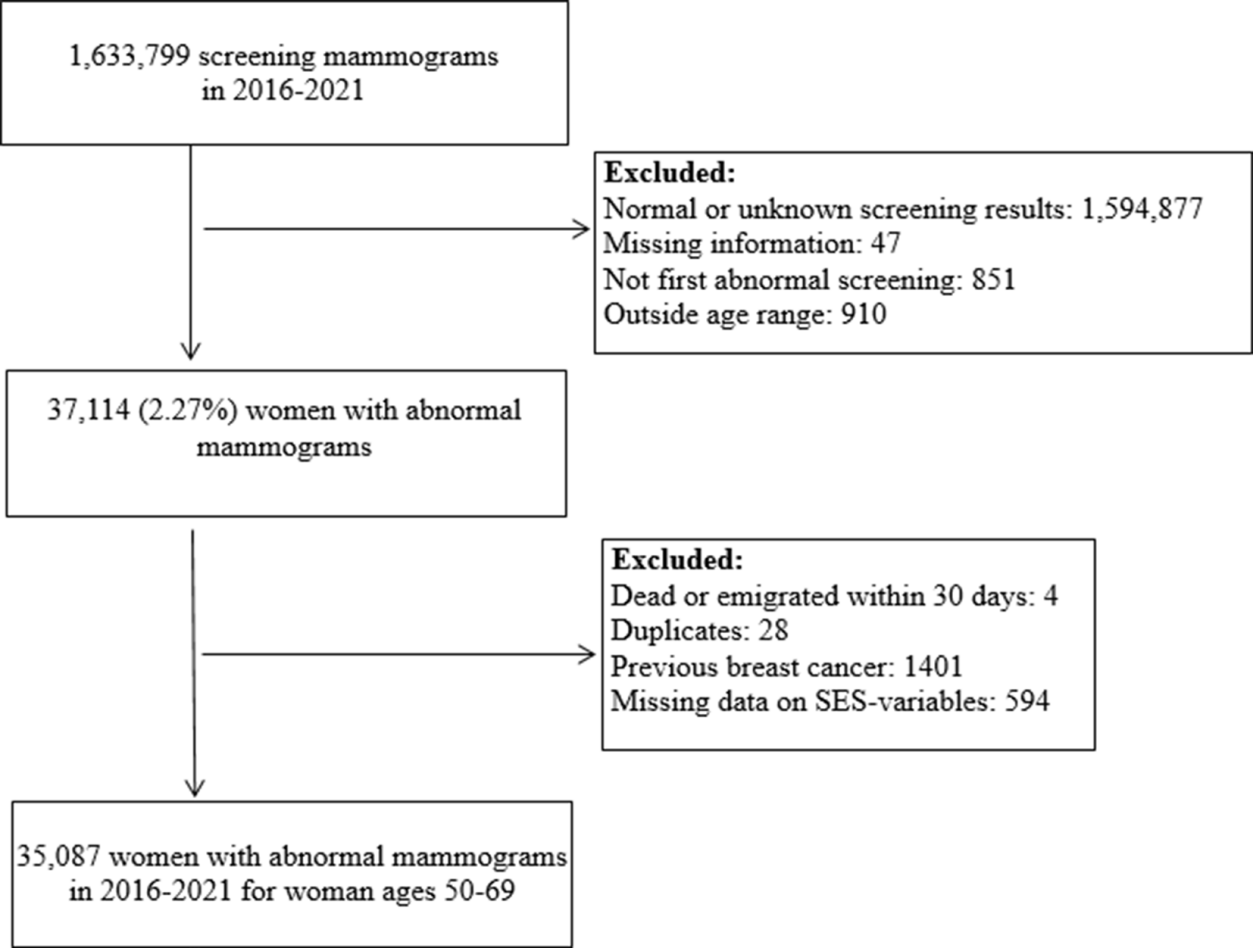
Flowchart showing the exclusions in the study

## Results

### Study population

Among 1,633,799 screening mammograms performed over six years, 37,114 were abnormal (referral rate: 2.27%), excluding subsequent abnormalities and missing results. After further exclusions, 35,087 women remained in the study population (Fig. 1).

Of these, 99.3% (34,825) had at least one follow-up procedure within six months, and 96.2% (33,508) received diagnostic procedures in line with national guidelines. Only 3.8% had less follow-up than recommended (Table 1).

**Table 1:** Socio demographic and economic characteristics of the study population by diagnostic follow-up pathways

	No follow-up	Mam./UL/biopsy	Mammo-graphy & UL	Less follow-up than expected	Total	*P*-value
***n*** ** (%)**	262 (0.7)	15,298 (43.6)	18,210 (51.9)	1317 (3.8)	35,087 (100.0)	
**Age group**,** n (%)**						
49–54	99 (0.7)	5344 (37.4)	8313 (58.1)	542 (3.8)	14,298 (100.0)	
55–59	58 (0.9)	2842 (42.3)	3564 (53.0)	255 (3.8)	6719 (100.0)	
60–64	49 (0.7)	3259 (48.8)	3136 (46.9)	241 (3.6)	6685 (100.0)	
65–70	56 (0.8)	3853 (52.2)	3197 (43.3)	279 (3.8)	7385 (100.0)	0.00
**Administrative region**,** n (%)**						
Capital Region	74 (0.8)	4016 (43.7)	4984 (54.3)	107 (1.2)	9181 (100.0)	
Central Denmark Region	61 (0.8)	3490 (47.1)	3436 (46.4)	420 (5.7)	7407 (100.0)	
North Denmark Region	43 (1.0)	1582 (38.0)	2360 (56.7)	174 (4.2)	4159 (100.0)	
Region Zealand	49 (0.9)	2600 (48.6)	2631 (49.2)	67 (1.3)	5347 (100.0)	
Region of Southern Denmark	35 (0.4)	3610 (40.1)	4799 (53.4)	549 (6.1)	8993 (100.0)	0.00
**Educational level**,** n (%)**						
10 years or less	78 (1.1)	3362 (45.7)	3595 (48.8)	327 (4.4)	7362 (100.0)	
Upper sec/vocational/Short edu(11–14 yrs)	122 (0.7)	7284 (43.2)	8828 (52.3)	636 (3.8)	16,870 (100.0)	
Bachelor or equivalent(15–17 yrs)	49 (0.6)	3496 (42.8)	4345 (53.1)	285 (3.5)	8175 (100.0)	
Master or more(> 17 yrs)	13 (0.5)	1156 (43.1)	1442 (53.8)	69 (2.6)	2680 (100.0)	0.00
**Wealth**,** n (%)**						
Level 1	57 (1.7)	1577 (45.8)	1663 (48.3)	147 (4.3)	3444 (100.0)	
Level 2	45 (0.7)	3045 (44.2)	3517 (51.0)	289 (4.2)	6896 (100.0)	
Level 3	76 (0.7)	4542 (43.2)	5483 (52.2)	402 (3.8)	10,503 (100.0)	
Level 4	84 (0.6)	6134 (43.1)	7547 (53.0)	479 (3.4)	14,244 (100.0)	0.00
**Employment**,** n (%)**						
Employed	135 (0.6)	9165 (41.0)	12,250 (54.8)	806 (3.6)	22,356 (100.0)	
Retired	51 (0.8)	3472 (52.0)	2915 (43.7)	234 (3.5)	6672 (100.0)	
Unemployed	76 (1.3)	2661 (43.9)	3045 (50.3)	277 (4.6)	6059 (100.0)	0.00
**Cohabitation status**,** n (%)**						
Cohabiting	167 (0.7)	10,736 (43.0)	13,117 (52.5)	969 (3.9)	24,989 (100.0)	
Living alone	95 (0.9)	4562 (45.2)	5093 (50.4)	348 (3.4)	10,098 (100.0)	0.00
**Country of origin**,** n (%)**						
Native	219 (0.7)	14,222 (43.6)	16,923 (51.9)	1237 (3.8)	32,601 (100.0)	
Western	12 (1.2)	441 (44.3)	516 (51.8)	27 (2.7)	996 (100.0)	
Non western	31 (2.1)	635 (42.6)	771 (51.7)	53 (3.6)	1490 (100.0)	0.00
**Severity of comorbidity**,** n (%)**						
None	221 (0.8)	12,552 (43.2)	15,259 (52.5)	1027 (3.5)	29,059 (100.0)	
Mild	34 (0.7)	2302 (45.1)	2537 (49.7)	233 (4.6)	5106 (100.0)	
moderate to severe	7 (0.8)	444 (48.2)	414 (44.9)	57 (6.2)	922 (100.0)	0.00

**Table 2:** Risk of non-timely follow-up

	Crude RR	CI95%	*P*-value	Adjusted RR	CI95%	*p*-value
**Administrative region**						
Capital Region of Denmark	15.86	13.21–19.03	0.000	15.99^a^	13.32–19.19	0.000
Central Denmark Region	Ref	-	-	Ref	-	-
North Denmark Region	22.02	18.33–26.46	0.000	21.99^a^	18.29–26.42	0.000
Region Zealand	11.62	9.634–14.02	0.000	11.66^a^	9.666–14.07	0.000
Region of Southern Denmark	0.565	0.427–0.749	0.000	0.565^a^	0.426–0.748	0.000
**Highest educational level**						
10 years or less	0.946	0.882–1.015	0.120	1.000^b^	0.937–1.068	0.998
Upper sec./vocational/Short edu.(11–14 yrs)	Ref	-	-	Ref	-	-
Bachelor or equivalent(15–17 yrs)	1.034	0.987–1.124	0.114	1.050^b^	0.989–1.115	0.110
Master or more (> 17 yrs)	1.355	1.241–1.479	0.000	1.126^b^	1.036–1.224	0.005
**Wealth**						
Level 1	1.074	0.977–1.180	0.139	0.980^c^	0.890–1.079	0.680
Level 2	0.968	0.895–1.046	0.406	1.003^c^	0.934–1.078	0.936
Level 3	Ref	-	-	Ref	-	-
Level 4	1.103	1.037–1.174	0.002	0.988^c^	0.931–1.047	0.675
**Employment**						
Employed	Ref	-	-	Ref	-	-
Retired	0.921	0.859–0.988	0.021	0.856^d^	0.766–0.955	0.006
Unemployed	1.029	0.961–1.102	0.406	1.084^d^	1.009–1.165	0.027
**Cohabitation status**						
Cohabiting	Ref	-	-	Ref	-	-
Living alone	1.072	1.014–1.134	0.014	1.073^e^	1.014–1.135	0.014
**Country of origin**						
Native	Ref	-	-	Ref	-	-
Western	1.204	1.046–1.388	0.010	1.173^f^	1.019–1.351	0.027
Non western	1.417	1.274–1.576	0.000	1.445^f^	1.298–1.609	0.000
**Severity of comorbidity**						
None	Ref	-	-	Ref	-	-
Mild	0.946	0.877–1.019	0.143	0.947^g^	0.879–1.022	0.161
moderate to severe	0.894	0.754–1.060	0.200	0.897^g^	0.756–1.065	0.214

a=Adjusted for wealth, comorbidity and age. b=adjusted for age, origin and region. c = adjusted for employment, age, origin and region. d = adjusted for age, education and comorbidity. e = adjusted for age. f = adjusted for education and age. g = adjusted for age

The risk of non-timely first follow-up (> 30 days) varied significantly across administrative regions. Compared to the Central Denmark region, women in the Capital Region had a 16-fold higher risk (RR = 15.99, 95%CI: 13.32–19.19). Women in Northern Denmark had a 22-fold higher risk (RR = 21.99, 95%CI: 18.29–26.42), while women in Region Zealand had an almost 12-fold higher risk (RR = 11.66, 95%CI: 9.666–14.07). Country of origin also influenced the risk of non-timely follow-up, as women of Western origin have a 17.3% higher risk (RR = 1.173, 95%CI: 1.019–1.351), and women of non-Western origin have a 44.5% higher risk (RR = 1.445, 95%CI: 1.298–1.609) of non-timely follow-up. Smaller differences were observed for women living alone compared to cohabitating (RR = 1.073, 95%CI: 1.014–1.135). Unemployed women compared to employed (RR = 1.084, 95%CI: 1.009–1.165), and women with a master’s degree compared to women with 11–14 years of schooling (RR = 1.126, 95%CI: 1.036–1.224) (Table 2).

**Fig. 2:**
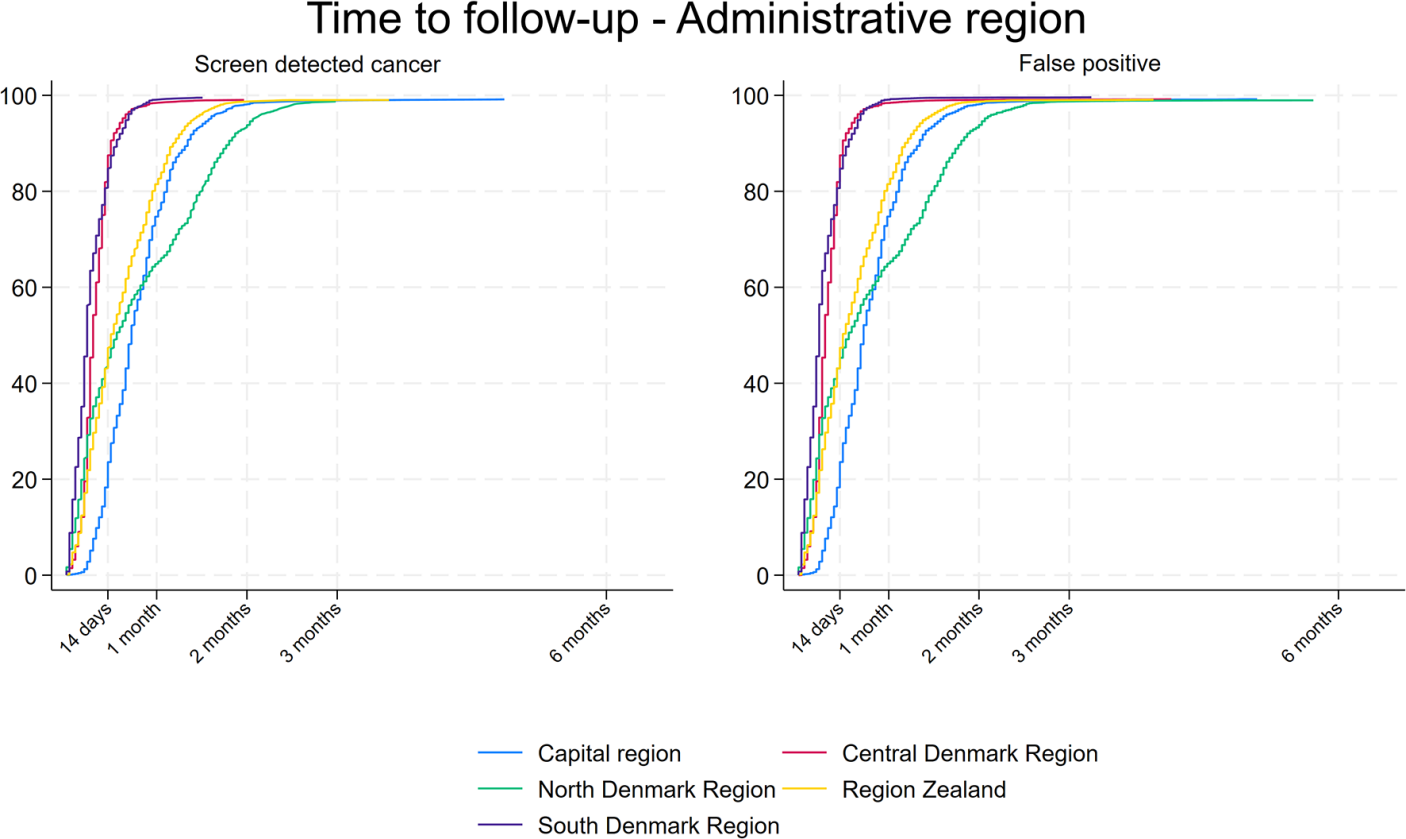
Time to first follow-up appointment distributed on administrative regions, grouped on SDC and false positive

Time to first follow-up varied greatly between regions (Fig. 2). However almost all women receive their first follow-up within 3 months. Time to first follow-up did not differ between women with SDC and women with false-positive results (Fig. 2).

When examining the risk of not receiving any follow-up within 6 months, we observed a general trend indicating that the highest risks were found among women with lower SEP, i.e. wealth level 1 (RR = 1.548, 95%CI: 1.043–2.299), less than 10 years of education (RR = 1.411, 95%CI: 1.058–1.881), unemployment (RR = 1.956, 95%CI: 1.043–2.299), living alone (RR = 1.408, 95%CI: 1.096–1.810) and being immigrant, both western (RR = 1.908, 95%CI: 1.069–3.404) and non-western (RR = 2.911, 95%CI: 1.991–4.256) (Table 3).

Women living in the Southern Denmark had 53.9% lower risk (RR = 0.461, 95%CI: 0.305–0.698) of not receiving any follow-up compared to women in the Central Denmark region. In the other regions, differences were also noted, although not statistically significant (Table 3).

The risk of having a biopsy performed as part of diagnostic follow-up among women not having SDC, was highest among women living in the Capital Region of Denmark (RR = 0.843, 95%CI: 0.794–0.894), Northern Denmark Region (RR = 0.716, 95%CI: 0.662–0.775) and Region of Southern Denmark (RR = 0.822, 95%CI: 0.775–0.873) compared to the Central Denmark region. Similarly, women with lower SEP and no breast cancer appeared significantly more likely to undergo a biopsy. This trend was observed among women in the lowest wealth level (RR = 1.122, 95%CI: 1.039–1.213), unemployed (RR = 1.123, 95%CI: 1.062–1.187), living alone (RR = 1.106, 95%CI: 1.058–1.156), women of non-Western origin (RR = 1.196, 95%CI: 1.096–1.305) and women with comorbidities—both mild (RR = 1.084, 95%CI: 1.022–1.149) and moderate to severe (RR = 1.303, 95%CI: 1.161–1.462) (Table 4)

**Table 3:** Risk of no follow-up

	Crude RR	CI95%	*P*-value	Adjusted RR	CI95%	*p*-value
**Administrative region**						
Capital Region of Denmark	0.979	0.698–1.372	0.901	1.030^a^	0.734–1.446	0.864
Central Denmark Region	Ref	-	-	Ref	-	-
North Denmark Region	1.255	0.851–1.851	0.251	1.231^a^	0.835–1.815	0.294
Region Zealand	1.113	0.765–1.618	0.576	1.106^a^	0.761–1.608	0.598
Region of Southern Denmark	0.473	0.312–0.715	0.000	0.461^a^	0.305–0.698	0.000
**Highest educational level**						
10 years or less	1.454	1.104–1.944	0.008	1.411^b^	1.058–1.881	0.019
Upper sec./vocational/Short edu.(11–14 yrs)	﻿Ref	-	-	﻿Ref	-	-
Bachelor or equivalent(15–17 yrs)	0.829	0.596–1.153	0.265	0.853^b^	0.613–1.187	0.346
Master or more (> 17 yrs)	0.671	0.379–1.187	0.170	0.649^b^	0.365–1.154	0.141
**Wealth**						
Level 1	2.287	1.626–3.218	0.000	1.548^c^	1.043–2.299	0.030
Level 2	0.902	0.625–1.302	0.581	0.831^c^	0.573–1.206	0.331
Level 3	﻿Ref	-	-	﻿Ref	-	-
Level 4	0.815	0.598–1.110	0.195	0.823^c^	0.601–1.127	0.224
**Employment**						
Employed	﻿Ref	-	-	﻿Ref	-	-
Retired	1.266	0.918–1.745	0.150	1.271^d^	0.748–2.162	0.375
Unemployed	2.077	1.570–2.747	0.000	1.956^d^	1.454–2.632	0.000
**Cohabitation status**						
Cohabiting	﻿Ref	-	-	﻿Ref	-	-
Living alone	1.408	1.095–1.809	0.008	1.408^e^	1.096–1.810	0.008
**Country of origin**						
Native	﻿Ref	-	-	﻿Ref	-	-
Western	1.794	1.007–3.196	0.047	1.908^f^	1.069–3.404	0.029
Non western	3.097	2.134–4.495	0.000	2.911^f^	1.991–4.256	0.000
**Severity of comorbidity**						
None	﻿Ref	-	-	﻿Ref	-	-
Mild	0.876	0.611–1.255	0.469	0.868^g^	0.605–1.246	0.442
moderate to severe	0.998	0.472–2.112	0.996	0.987^g^	0.465–2.093	0.973

a=Adjusted for wealth, comorbidity and age. b=adjusted for age, origin and region. c = adjusted for employment, age, origin and region. d = adjusted for age, education and comorbidity. e = adjusted for age. f = adjusted for education and age. g = adjusted for age

**Table 4:** Risk of having a biopsy performed among women with false positive screening result

	Crude RR	CI95%	*P*-value	Adjustet RR	CI95%	*p*-value
**Geographic region**						
Capital Region of Denmark	0.832	0.785–0.883	0.000	0.843^a^	0.794–0.894	0.000
Central Denmark Region	﻿Ref	-	-	﻿Ref	-	-
North Denmark Region	0.751	0.666–0.781	0.000	0.716^a^	0.662–0.775	0.000
Region Zealand	0.995	0.933–1.061	0.873	0.997^a^	0.935–1.062	0.923
Region of Southern Denmark	0.829	0.781–0.880	0.000	0.822^a^	0.775–0.873	0.000
**Highest educational level**						
10 years or less	1.012	0.958–1.068	0.678	1.033^b^	0.977–1.091	0.255
Upper sec./vocational/Short edu.(11–14 yrs)	﻿Ref	-	-	﻿Ref	-	-
Bachelor or equivalent(15–17 yrs)	0.973	0.923–1.035	0.303	0.987^b^	0.937–1.040	0.628
Master or more (> 17 yrs)	0.994	0.919–1.076	0.888	0.998^b^	0.921–1.091	0.954
**Wealth**						
Level 1	1.180	1.100-1.265	0.000	1.122^c^	1.039–1.213	0.003
Level 2	1.059	1.000-1.123	0.052	1.048^c^	0.988–1.111	0.119
Level 3	﻿Ref	-	-	﻿Ref	-	-
Level 4	0.913	0.867–0.960	0.000	0.933^c^	0.886–0.982	0.008
**Employment**						
Employed	﻿Ref	-	-	Ref	-	-
Retired	0.966	0.910–1.025	0.257	1.085^d^	0.980–1.201	0.117
Unemployed	1.123	1.065–1.183	0.000	1.123^d^	1.062–1.187	0.000
**Cohabitation status**						
Cohabiting	Ref	-	-	Ref	-	-
Living alone	1.104	1.056–1.154	0.000	1.106^e^	1.058–1.156	0.000
**Country of origin**						
Native	Ref	-	-	Ref	-	-
Western	1.032	0.913–1.167	0.610	1.027^f^	0.908–1.161	0.673
Non western	1.233	1.131–1.344	0.000	1.196^f^	1.096–1.305	0.000
**Severity of comorbidity (without diabetes)**						
none	Ref	-	-	Ref	-	-
mild	1.061	1.000-1.124	0.045	1.084^g^	1.022–1.149	0.007
moderate to severe	1.253	1.115–1.406	0.000	1.303^g^	1.161–1.462	0.000

a=Adjusted for wealth, comorbidity and age. b=adjusted for age, origin and region. c = adjusted for employment, age, origin and region. d = adjusted for age, education and comorbidity. e = adjusted for age. f = adjusted for education and age. g = adjusted for age

## Discussion

### Main findings

In Denmark, 99.3% of women with abnormal screening mammograms had at least one diagnostic follow-up procedure. Among those 96.2% had diagnostic procedures in accordance with the national recommendations.

SEP variables were associated with adherence to follow-up in relation to both the timeliness and adequacy of follow-up. Despite mandated cancer care pathways, women with lower SEP, immigrants, unemployed women, and women with severe comorbidities were more likely to experience non-timely or less follow-up than recommended, in several, but not all, analyses depending on the specific socioeconomic indicator and outcome. There were significant regional differences in the timeliness and adequacy of follow-up after an abnormal screening mammogram in Denmark. Women in the Capital-, Northern Denmark-, and Region Zealand faced a substantially higher risk of non-timely follow-up compared to the other two regions. Women in Northern Denmark and Region Zealand also had a non-significantly higher risk of not having follow-up at all.

### Strength and limitations

In Denmark high-quality register data is widely available, enabling us to use register data on individual level with complete follow-up.

However, this study still have some limitations. In Denmark, women with an abnormal mammography are recommended to have the diagnostic mammography performed in National Breast Centers [20]. Some women may have these procedures performed at private hospitals, either by choice or because public hospitals refer them due to extended waiting times. Use of private healthcare may be more common among women with higher SEP, potentially leading to differential capture of follow-up across socioeconomic groups. As data from private hospitals are less complete in the Danish National Patient Registry [21], follow-up may be underestimated, particularly in higher SEP groups. However, private hospitals accounted for only 4% of hospital activity in 2014 and 7% in 2022, and are primarily used for conditions other than cancer diagnostics [31]. Excluding 594 women (1.7%) through the complete-case approach may have slightly affected the composition of the study population, as these women were more often immigrants and lacked educational information. Overall, 4% had no follow-up and 20% experienced non-timely follow-up, indicating that this excluded group likely represents a particularly non-adherent population.

The classification of follow-up pathways as “appropriate” or “less than expected” was based on predefined procedure combinations from national recommendations and registry data. While this ensures standardized and reproducible definitions, it may not fully reflect real-world clinical flexibility, where pathways are tailored to patient characteristics, clinical judgment, and organizational factors. Consequently, some pathways labelled “less than expected” may represent appropriate care rather than suboptimal management. However, this operationalization was necessary to enable consistent classification across a nationwide dataset and facilitate comparisons across socioeconomic groups and regions.

Finally, the interpretation of socioeconomic differences should consider that associations are reference-group dependent and vary across SEP variables. Reference categories were chosen based on representativeness rather than being consistently the highest or lowest SEP groups.

To ensure that previous abnormal findings did not influence clinical decisions, we conducted a sensitivity analysis that adjusted for prior abnormal findings before the study period. The adjustment did not affect the results. Similarly, we performed a sensitivity analysis were the study population was divided into those screened 2016–2019 and those screened 2020–2021, to examine whether the COVID-19 lockdown could have influenced the outcomes. No significant differences were observed between the two groups.

### Comparison with the literature and clinical implications

Significant regional variations in follow-up timeliness were observed in this study, with longer delays particularly evident in the Capital-, Northern Denmark-, and Region Zealand. While the majority of women received timely follow-up, a substantial proportion (14.2%) experienced delays exceeding 30 days (Supplementary, table S1). Median time to first follow-up was relatively short—11 days for women with SDC and 13 days for those with false-positive screening results—yet time to full resolution varied considerably across regions (Supplementary, table S1, Fig. S1). These differences suggest that structural factors—such as variations in healthcare capacity, workforce distribution, and administrative procedures—may contribute to disparities in the efficiency of diagnostic pathways.

The continued presence of regional variation, even after adjusting for relevant SEP variables, further indicates underlying inequalities in resource allocation, administrative efficiency, or available capacity. Our previous study found similar time to first follow-up and time to resolution, examining the same screening program but over an earlier time period indicating that these challenges persist over time [32], with the addition of Northern Denmark Region now also performing poorly in terms of timeliness, unlike in the previous study.

This is also supported by the data presented in the annual report from the Danish Quality Database for Mammography Screening. Indicator 10, concerning the response time for screening mammography, has not been consistently met in the Capital Region of Denmark, Region Zealand, and North Denmark Region across several years during the study period [33–35]. When the screening result is non-timely, it may be difficult to ensure that diagnostic work-up is initiated within 30 days. The report even states that not all women in North Denmark Region received their screening results within 30 days [35]. The yearly reports from the Danish Quality Database for Mammography screening have shown that 99% of women get a diagnostic follow-up within 2 months [33–35]. Both of these indicators of the report appears in line with our results.

While Southern and Central Denmark Regions perform well in terms of timeliness, they present the highest risk of missing elements during follow-up (Supplementary, table S1). These differences can be caused by region-specific healthcare practices, or workforce capacities. Moreover, local referral patterns and organizational structures may influence the delivery of follow-up care—for instance, a stronger tendency to utilize already available mammography may reduce the need for additional examinations and limit radiation exposure for the woman. To examine whether such differences could affect our results, we conducted an additional analysis, categorizing women with ultrasound and biopsy as in accordance with recommendations. This revealed no significant changes.

Another finding, indicating local follow-up practices, was that the likelihood of performing a biopsy for women with false positive screenings result was higher in some regions compared to others. This may be related to the general lack of radiologists and radiographists, which may be unequally distributed between regions [36, 37]. Such regional differences in follow-up practices may reflect local protocols or regional disparities in access to experienced radiologists, as less experienced radiologists may be more likely to do a reassuring biopsies. However, we do not have data to support that.

Besides these structural inequalities, based on region of residence, women with lower SEP —including shorter education (< 10 years), low wealth, and unemployment— were more likely to experience non-timely or less follow-up than recommended across several analyses, although the magnitude and consistency of associations varied across SEP variables and outcomes. These women appear particularly vulnerable in the diagnostic pathways following abnormal screening results in breast cancer programs [8, 10]. The overlap between low SEP and limited health literacy may be associated with greater difficulties navigating the healthcare system and advocate for appropriate follow-up care. Immigrant women face both an increased risk of no follow-up and non-timelyfollow-up compared to natives, a pattern that mirrors previous findings showing lower screening participation among immigrants [7]. This suggests that barriers such as language, cultural differences, and limited health literacy continues to play a role, even among those who attend their screening appointments [38].

Considering the prolonged response time of the screening program, women with low SEP may also be more likely to reschedule their initial appointments, due to a limited understanding of the importance of timely follow-up or the need for support—such as someone to accompany or transport them to the appointment. If the response time is already close to the 30-day threshold, even minor delays could result in a failure to meet the recommended timeframe. Additionally, large regional differences may further widen existing disparities, leaving those with lower SEP or health literacy at an even greater disadvantage.

Lastly, women with comorbidities, another potentially vulnerable group, were at greater risk of less follow-up than recommended, suggesting that pre-existing health conditions may interfere with diagnostic procedures. Interestingly, our analyses showed that these women were also more likely to undergo biopsies as part of the diagnostic pathway. This paradox may stem from the complexities of managing patients with multiple health conditions in regular contact with the health care system. Thus, both under- and overtreatment can be of concern.

This study highlights significant regional and socioeconomic disparities in follow-up care for women with abnormal mammography results in Denmark. While most women receive timely diagnostic follow-up, notable variations persist across administrative regions in both the timeliness and completeness of care. Women from lower SEP backgrounds, with higher comorbidity burden and non-Western immigrant groups, were more likely to experience non-timely or less follow-up than recommended. These women have expressed their interest in screening by participation. Addressing these inequalities is important for developing equitable screening strategies and ensuring consistent, unbiased care delivery across socioeconomic groups, even within structured cancer pathways.

## Supplementary Information

Below is the link to the electronic supplementary material.

Supplementary Material 1

## Data Availability

No datasets were generated or analysed during the current study.
