# Supplementary material for: Socioeconomic variation in adherence to follow-up after an abnormal screening mammogram in the Danish breast cancer screening program
Source: Breast Cancer Res Treat. 2026 Jul 9;218(1):5. doi: 10.1007/s10549-026-08014-3 (PMC13350214; doi:10.1007/s10549-026-08014-3)
Supplement: Supplementary file 1 — Supplementary Material 1 [file 10549_2026_8014_MOESM1_ESM.docx]

**Supplementary**

**Table S1:** Risk of insufficient follow-up

**Figure S1:** Time to resolution by region

**Table S2:** Median time to follow-up and resolution

|  | **Crude RR** | **CI95%** | **P-value** | **Adjusted RR** | **CI95%** | **p-value** |
| --- | --- | --- | --- | --- | --- | --- |
| **Administrative region** |  |  |  |  |  |  |
| Capital region | 0.205 | 0.167-0.254 | 0.000 | 0.205^a^ | 0.166-0.252 | 0.000 |
| Central Denmark Region | 1 | - | - | 1 | - | - |
| North Denmark Region | 0.739 | 0.622-0.879 | 0.001 | 0.743^a^ | 0.625-0.883 | 0.000 |
| Region Zealand | 0.221 | 0.171-0.286 | 0.000 | 0.219^a^ | 0.170-0.283 | 0.001 |
| Southern Denmark Region | 1.072 | 0.948-1.213 | 0.269 | 1.071^a^ | 0.946-1.211 | 0.000 |
| **Highest educational level** |  |  |  |  |  |  |
| 10 years or less | 1.182 | 1.038-1.347 | 0.012 | 1.123^b^ | 0.984-1.281 | 0.084 |
| Upper sec./vocational/Short edu.(11-14 yrs) | 1 | - | - | 1 | - | - |
| Bachelor or equivalent(15-17 yrs) | 0.924 | 0.805-1.059 | 0.256 | 0.936^b^ | 0.816-1.072 | 0.339 |
| Master or more (>17 yrs) | 0.681 | 0.533-0.870 | 0.002 | 0.854^b^ | 0.669-1.091 | 0.207 |
| **Wealth** |  |  |  |  |  |  |
| Level 1 | 1.126 | 0.936-1.354 | 0.209 | 1.061^c^ | 0.868-1.299 | 0.562 |
| Level 2 | 1.094 | 0.944-1.269 | 0.234 | 1.043^c^ | 0.898-1.211 | 0.582 |
| Level 3 | 1 | - | - | 1 | - | - |
| Level 4 | 0.877 | 0.944-1.269 | 0.049 | 1.014^c^ | 0.889-1.155 | 0.840 |
| **Employment** |  |  |  |  |  |  |
| Employed | 1 | - | - | 1 | - | - |
| Retired | 0.974 | 0.845-1.124 | 0.722 | 0.802^d^ | 0.639-1.008 | 0.058 |
| Unemployed | 1.276 | 1.117-1.459 | 0.000 | 1.137^d^ | 0.987-1.310 | 0.075 |
| **Cohabitation status** |  |  |  |  |  |  |
| Cohabiting | 1 | - | - | 1 | - | - |
| Living alone | 0.891 | 0.790-1.005 | 0.060 | 0.891^e^ | 0.790-1.005 | 0.060 |
| **Country of origin** |  |  |  |  |  |  |
| Native | 1 | - | - | 1 | - | - |
| Western | 0.718 | 0.493-1.046 | 0.912 | 0.747^f^ | 0.513-1.088 | 0.129 |
| Non western | 0.951 | 0.726-1.246 | 0.664 | 0.904^f^ | 0.689-1.186 | 0.467 |
| **Severity of comorbidity** |  |  |  |  |  |  |
| none | 1 | - | - | 1 | - | - |
| mild | 1.290 | 1.222-1.482 | 0.000 | 1.299^g^ | 1.130-1.495 | 0.000 |
| moderate to severe | 1.748 | 1.351-2.265 | 0.000 | 1.771^g^ | 1.366-2.297 | 0.000 |

***Table S1****: Risk of getting less than expected follow-up. a=Adjusted for wealth, comorbidity and age. b=adjusted for age, origin and region. c = adjusted for employment, age, origin and region. d = adjusted for age, education and comorbidity. e = adjusted for age. f = adjusted for education and age. g = adjusted for age*


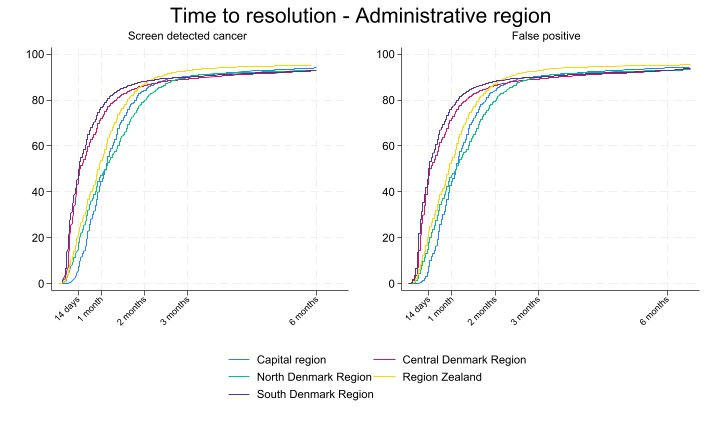


**Fig. S1** *Time to resolution distributed on administrative regions, grouped on screen detected cancer and false positive*

|  | **N (%)** | **Screen detected breast cancers**  *(N, %)* | **False positive screenings result**  *(N, %)* | **Time to first follow-up appointment** *(median, IQR)* | | **Time to resolution**  *(median, IQR)* | |
| --- | --- | --- | --- | --- | --- | --- | --- |
|  |  |  |  | **Cancer** | **FP** | **Cancer** | **FP** |
| Total | 35087 (100.0) | 8646 (25,5) | 26141 (74,5) | 11 (7; 20) | 13 (7; 22) | 37 (27; 53) | 19 (11; 35) |
| **Geographic region** |  |  |  |  | |  | |
| Capital region | 9181 (26.2) | 2374 (25.9) | 6807 (74.1) | 19 (13; 27) | 23 (16; 32) | 44 (35; 57) | 28 (20; 42) |
| Central Denmark Region | 7407 (21.1) | 2065 (27.9) | 5342 (72.1) | 9 (7; 12) | 9 (7; 13) | 30 (24; 42) | 12 (8; 20) |
| North Denmark Region | 4159 (11.9) | 931 (22.4) | 3228 (77.6) | 17 (6; 38) | 17 (7; 42) | 45 (29; 65) | 28 (15; 50) |
| Region Zealand | 5347 (15.2) | 1486 (27.8) | 3861 (72.2) | 16 (8; 27) | 14 (8; 25) | 42 (32; 58) | 22 (13; 35) |
| Southern Denmark Region | 8993 (25.6) | 2090 (23.2) | 6903 (76.8) | 7 (4; 12) | 7 (3; 11) | 29 (22; 41) | 12 (7; 20) |
| **P-value** |  | 0.000 | | 0.0001 | 0.0001 | 0.0001 | 0.0001 |
| **Highest educational level** |  |  |  |  | |  | |
| 10 years or less | 7362 (21.0) | 2101 (28.5) | 5261 (71.5) | 10 (6; 18) | 12 (7; 21) | 35 (26; 51) | 18 (10; 35) |
| Upper sec./vicational/Short edu.(11-14 yrs) | 16870 (48.1) | 4172 (24.7) | 12698 (75.3) | 11 (7; 19) | 13 (7; 22) | 36 (27; 53) | 19 (11; 35) |
| Bachelor or equiavalent(15-17 yrs) | 8175 (23.3) | 2022 (24.7) | 6153 (75.3) | 12 (7; 20) | 13 (7; 23) | 37 (27; 52) | 19 (10; 36) |
| Master or more (>17 yrs) | 2680 (7.6) | 651 (24.3) | 2029 (75.7) | 13 (8; 24) | 16 (9; 27) | 41 (30; 58) | 22 (13; 38) |
| **P-value** |  | 0.000 | | 0.0001 | 0.0001 | 0.0001 | 0.0001 |
| **Wealth** |  |  |  |  | |  | |
| 1st quartile (<25%) | 3444 (9.8) | 828 (24.0) | 2616 (76.0) | 11 (7; 20) | 13 (7; 22) | 37 (27; 55) | 20 (11; 39) |
| 2nd quartile (25-50%) | 6896 (19.7) | 1708 (24.8) | 5188 (75.2) | 11 (6; 19) | 12 (7; 21) | 36 (26; 51) | 18 (10; 36) |
| 3rd quartile (50-75%) | 10503 (29.9) | 2615 (24.9) | 7888 (75.1) | 10 (6; 19) | 12 (7; 21) | 35 (26; 51) | 18 (10; 35) |
| 4th quartile (>75%) | 14244 (40.6) | 3795 (26.6) | 10449 (73.4) | 12 (7; 20) | 14 (7; 24) | 38 (28; 54) | 20 (11; 35) |
| **P-value** |  | 0.001 | | 0.0001 | 0.0001 | 0.0001 | 0.0004 |
| **Employment** |  |  |  |  | |  | |
| Employed | 22356 (63.7) | 4968 (22.2) | 17388 (77.8) | 11 (7; 20) | 13 (7; 23) | 37 (27; 53) | 19 (11; 35) |
| Unemployed | 6059 (17.3) | 1430 (23.6) | 4629 (76.4) | 11 (7; 19) | 12 (7; 22) | 37 (27; 54) | 19 (11; 37) |
| Retired | 6672 (19.0) | 2548 (38.2) | 4124 (61.8) | 11 (7; 19) | 12 (7; 21) | 36 (27; 52) | 17 (10; 35) |
| **P-value** |  | 0.000 | | 0.5630 | 0.0001 | 0.2397 | 0.0013 |
| **Cohabition status** |  |  |  |  | |  | |
| Cohabiting | 24989 (71.2) | 6361 (25.5) | 18628 (74.5) | 11 (7; 19) | 12 (7; 22) | 36 (27; 52) | 19 (10; 35) |
| Living alone | 10098 (28.8) | 2585 (25.6) | 7513 (74.4) | 12 (7; 20) | 13 (7; 24) | 38 (27; 54) | 20 (11; 37) |
| **P-value** |  | 0.779 | | 0.0008 | 0.0001 | 0.0220 | 0.0001 |
| **Country of origin** |  |  |  |  | |  | |
| Native | 32601 (92.9) | 8418 (25.8) | 24183 (74.2) | 11 (7; 20) | 13 (7; 22) | 36 (27; 52) | 19 (10; 35) |
| Western | 996 (2.8) | 253 (25.4) | 743 (74.6) | 10 (7; 21) | 14 (7; 26) | 38 (27; 55) | 21 (12; 39) |
| Non western | 1490 (4.2) | 275 (18.5) | 1215 (81.5) | 13 (7; 21) | 15 (8; 27) | 42 (31; 63) | 24 (14; 44) |
| **P-value** |  | 0.000 | | 0.0062 | 0.0001 | 0.0062 | 0.0001 |
| **Severity of comorbidity (without diabetes)** |  |  |  |  | |  | |
| none | 29059 (82.8) | 7334 (25.2) | 21725 (74.8) | 11 (7; 20) | 13 (7; 22) | 36 (27; 53) | 19 (11; 35) |
| mild | 5106 (14.6) | 1361 (26.7) | 3745 (73.3) | 12 (7; 20) | 13 (7; 23) | 37 (28; 51) | 20 (11; 36) |
| moderate to severe | 922 (2.6) | 251 (27.2) | 671 (72.8) | 13 (7; 21) | 12 (7; 21) | 37 (28; 54) | 20 (11; 40) |
| **P-value** |  | 0.048 | | 0.0757 | 0.2849 | 0.6052 | 0.0460 |

**Table S2** *overview of median time to first follow-up appointment and time to resolution.*
